# Facteurs liés au diagnostic tardif du cancer du sein: expérience du CHU Mohammed VI Marrakech

**DOI:** 10.11604/pamj.2015.21.162.4363

**Published:** 2015-06-25

**Authors:** Sofia Aloulou, Amal El Mahfoudi, Abdelhamid El Omrani, Mouna Khouchani

**Affiliations:** 1 Service d'Oncologie Radiothérapie, CHU Mohammed VI Marrakech, Maroc

**Keywords:** Cancer du sein, retard diagnostic, santé publique, breast cancer, diagnostic delay, public health

## Abstract

Le cancer du sein est le premier cancer féminin en termes d'incidence et de mortalité. Au Maroc, il vient au premier rang des cancers de la femme avant celui du col utérin Il constitue un problème de santé publique. Son pronostic est étroitement lié au stade auquel le diagnostic est posé. Il s'agit d'une pathologie dont les moyens diagnostiques sont de nos jours développés, allant de la détection précoce à la mise en évidence de lésions infra-cliniques, ce qui a nettement amélioré le pronostic dans les pays développés. Ce travail que nous présentons a pour objectif d'identifier dans notre pratique quotidienne, les facteurs qui amènent les patientes à consulter à des stades tardifs. Une étude rétrospective a été menée de janvier 2012 à janvier 2013 portant sur 130 patientes porteuses d'un cancer du sein au sein du service d'onco-radiotherapie CHU Mohammed VI Marrakech. Un questionnaire a été élaboré et dument renseigné en ayant recours aux dossiers des malades. Ainsi 63,07% des patientes consultaient au-delà de six mois avec un délai moyen de consultation de 8,47 mois avec comme motif de consultation des lésions classées T4 dans 27,69%, et des tumeurs d'emblée métastatiques dans 13,84%. Les facteurs retrouvés à l'interrogatoire étaient le manque de moyens financiers 40%, l’éloignement des structures sanitaires dans 23%, les habitudes socioculturelles avec les traitements traditionnels en première intention 20%, et l'insuffisance de prise en charge thérapeutique 7%. Cependant, pris individuellement, aucune concordance significative n’était retrouvée entre ces facteurs et le long délai diagnostique. Dans notre pratique, c'est la conjonction de la triade ignorance, indigence et habitudes socioculturelles qui constituent le facteur essentiel du diagnostic tardif des cancers du sein.

## Introduction

Le cancer du sein représente un véritable problème de santé publique majeur. Dans le Monde, 540 000 cas de cancer du sein apparaissent chaque année et près de 300 000 femmes en meurent, généralement à cause d'un dépistage tardif. Au Maroc, le cancer du sein est la tumeur la plus fréquente chez les femmes. Il représente la première cause de mortalité féminine par un cancer. Il frappe chaque année entre 13.000 et 30.000 marocaines, a indiqué l'Association Lalla Salma de lutte contre le cancer (ALSC) [1–3]. Il constitue un problème de santé publique souvent considéré comme un événement dramatique dans la vie d'une femme. Son pronostic est étroitement lié au stade auquel le diagnostic est posé. Il s'agit d'une pathologie dont les moyens diagnostiques sont de nos jours développés, allant de la détection précoce à la mise en évidence de lésions infra-cliniques, ce qui a nettement amélioré le pronostic dans les pays développés. Au Maroc le diagnostic du cancer du sein est fait au stade I dans seulement 6% des cas contre 57% des cas au stade III et IV [2, 3]. Ce travail que nous présentons a pour objectif d'identifier dans notre pratique quotidienne, les facteurs qui amènent les patientes à consulter à des stades tardifs.

## Méthodes

Une étude rétrospective a été menée de janvier 2012 à janvier 2013 portant sur 130 patientes porteuses d'un cancer du sein confirmé au sein du service d'onco-radiotherapie CHU Mohammed VI Marrakech. Pour la réalisation de ce travail, nous avons élaboré un questionnaire comprenant les différentes variables nécessaires à notre étude. Les questionnaires ont dûment été renseignés en ayant recours aux dossiers des malades. Le délai du diagnostique était défini comme étant la période entre la perception des premiers signes par la patiente et la date de confirmation histologique. Nous avons recherché les circonstances de découverte de ces cancers: nodule, inflammation, ulcération, métastases révélatrices. Les potentiels facteurs influençant le retard diagnostique ont été analysés: traitements traditionnels antérieurs, peur du diagnostic, erreurs diagnostiques, l'insuffisance de prise en charge médicale. Les traitements traditionnels ont consisté en des pratiques non médicales, mise en œuvre par des personnes non assermentées pour le traitement des cancers du sein. La peur du diagnostic décrivait une appréhension du diagnostic de cancer qui a conduit la patiente, devant une symptomatologie mammaire, à différer a maintes reprises la consultation dans un centre spécialisé. Ont été considérées comme erreurs diagnostiques toutes les patientes présentant une forte présomption de cancer du sein, n'ayant bénéficié d'aucun examen histologique mais traité depuis plus de six mois pour une pathologie autres que le cancer du sein (granulomatose mammaire, tuberculose, abcès du sein). Quant à l'insuffisance de prise en charge thérapeutique, elle traduisait la mise en œuvre d'une seule méthode thérapeutique là où le stade évolutif justifiait la conjonction de plusieurs méthodes notamment la chimiothérapie, la chirurgie et la radiothérapie. Les données cliniques et biologiques sont recueillies, codées, saisies sur Microsoft office Excel 2003, puis analysées au moyen du logiciel SPSS.

## Résultats

**Figure 1:**
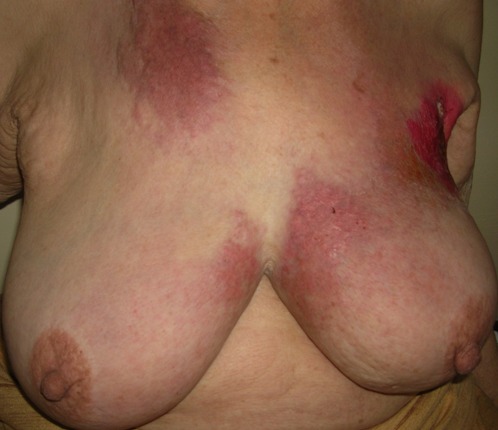
Cancer inflammatoire chez une patiente de 60 ans

**Figure 2:**
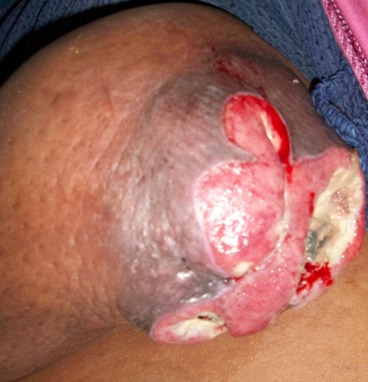
Cancer ulcéré chez une patiente de 40 ans

L′étude a porté sur 130 patientes atteintes d′un carcinome mammaire invasif confirmé à l'histologie. La population de cette région est caractérisée par un âge jeune, avec un âge médian de 46 ans, et des extrêmes allant de 20 à 78 ans. 63,07% des patientes consultaient au-delà de six mois avec un délai moyen de consultation de 8,47 mois avec comme motif de consultation des lésions classées T4 dans 27,69%: inflammation 11,54% (Figure 1), ulcération 16,15% (Figure 2), et des tumeurs d'emblée métastatiques dans 13,84%. Le nodule isolé du sein, constituait 58,46% des motifs. L′étude clinico-pathologique a montré que le diagnostic du cancer du sein est tardif, en effet la taille moyenne des tumeurs est de 3,5 cm avec 75% des cas qui ont une taille supérieure à 2 cm T2 et T3 (Tableau 1). Le type histologique prédominant des tumeurs est le carcinome canalaire infiltrant dans 90% des cas, suivi du carcinome lobulaire infiltrant (5%). Le nombre des tumeurs grade histologique II et III est élevé (56% et 28% respectivement).

**Tableau 1:** Rrépartition des patientes en fonction du délai diagnostique/circonstances de découverte

Circonstances de découverte	Délai diagnostic < 6 mois	Délai diagnostic 6 – 10 mois	Délai >10 mois	Total (n)	%
Nodule	40	18	18	76	58,46
Inflammation	2	5	8	15	11,54
Ulcération nécrotique	4	7	10	21	16,15
Métastases révélatrices	2	7	9	18	13,84
Effectif total (n)	48	37	45	130	100
%	36,93	28,46	34,61	100	

Concernant les facteurs identifiés pour justifier le long délai diagnostique, l'interrogatoire a mis en évidence, le manque de moyens financiers 40%, l’éloignement des structures sanitaires dans 23,07%, les habitudes socioculturelles avec les traitements traditionnels en première intention 20%, et l'insuffisance de prise en charge thérapeutique 7% et les erreurs diagnostiques 3%. Cependant, pris individuellement, aucune concordance significative n’était retrouvée entre ces facteurs et le long délai diagnostique (Tableau 2).

**Tableau 2:** Facteurs incriminés dans le long délai diagnostique

	Délai diagnostic < 6 mois	Délai diagnostic 6 – 10 mois	Délai >10 mois	Total n	%
Manque de moyens financiers	12	16	24	52	40
Eloignement	16	10	4	30	23
Habitudes socioculturelles (Traitements traditionnels)	12	8	6	26	20
Insuffisance de prise en charge médicale	4	3	2	9	7
Erreur diagnostique	2	0	6	8	6
Peur	2	0	3	5	4
Effectif total n (%)	48 (36,93%)	37 (28,46%)	45 (34,61%)	130	100

## Discussion

Le diagnostic tardif des cancers du sein demeure un problème d'actualité dans notre milieu d'exercice. Dans notre série comme dans la plupart des publications africaines, le délai diagnostique était particulièrement long (supérieur à dix mois), dans notre série le délai moyen est de 8,47 mois [1, 4, 5]. Les raisons du diagnostic tardif étaient multiples et dominées par les problèmes financiers. Egalement, l′accès géographique et économique aux unités de soins en oncologie est un vrai problème de santé public au Maroc, car le pays ne dispose actuellement que de 6 centres étatiques en oncologie. La prise en charge est aussi affectée à plusieurs niveaux: adhésion difficile au plan de soins, mauvaise observance ou interruption du traitement, patients perdus de vu [2, 3, 6].

Les habitudes socioculturelles représentées par une fréquentation en première intention de la médecine traditionnelle et le problème de qualification du personnel constituaient également des raisons pour un diagnostic tardif des cancers du sein. La formation du personnel soignant surtout les médecins généralistes, infirmiers, les sages-femmes (personnes les plus sollicitées en zone rurale) devrait pouvoir limiter les risques d'erreurs diagnostiques dans la mesure où le cancer du sein pose souvent un problème de diagnostic différentiel avec les mastopathies bénignes. [2, 3, 7, 8]. Le vécu psychique du patient est lourdement affecté, aux troubles psychologiques qui accompagnent habituellement toute annonce du cancer passant par l′acceptation de la maladie jusqu′au deuil; tels que la dépression, l′angoisse et la peur de la mort, vont se greffer d′autres troubles: dévalorisation ou perte d′estime de soi, isolement, troubles du schéma corporel et/ou de l′identité sexuelle, pouvant être aussi réprimandant que la maladie en elle-même. Le vécu social du patient qui fuira continuellement le regard des autres est une autre rude épreuve. [5, 9]. Cependant, aucun des facteurs incriminés, pris individuellement, n'a d'incidence sur le long délai diagnostique. C'est surtout la conjonction de tous ces facteurs qui concourait au diagnostic tardif chez nos patientes. Sur le plan clinique, l'interrogatoire dans notre série montrait que moins de 6% des patientes pratiquaient l'autopalpation qui représente un acte important dans le diagnostic précoce du cancer du sein. Il s'agissait plutôt d'une autopalpation fortuite au décours de signes évidents (pesanteur, douleur mammaire). En occident par contre, elle constitue avec le dépistage systématique un reflex habituel chez la femme, si bien que 80% des patientes sont vues à des stades précoces [10, 11]. Les circonstances de découverte dans notre série étaient dominées par des tumeurs de toute évidence suspectes de malignité à l'examen clinique avec de volumineuses masses plus ou moins associées à des signes inflammatoires voire même des ulcérations. Dans les pays développés, le diagnostic précoce prédominait au stade de nodules et de lésions infra-cliniques détectées par la mammographie de dépistage [10–12].

L'histoire naturelle du cancer du sein nous montre qu'il s'agit d'une maladie générale à expression locorégionale. Plus le délai de consultation est long, plus le risque métastatique est grand et la survie mauvaise. Dans notre pays l'ALSC joue un rôle important dans le domaine de la prévention et le traitement. Aujourd'hui, cette association enchaîne les campagnes de sensibilisation pour permettre une prise de conscience chez les femmes marocaines afin qu'elles aillent se faire dépistées au plus vite. En effet, le dépistage précoce est essentiel et permet une meilleure guérison.

## Conclusion

La triade ignorance, indigence et habitudes socioculturelles constituaient les facteurs essentiels du diagnostic tardif des cancers du sein. Le dépistage précoce passe par l'information, l’éducation et la lutte contre la pauvreté. Une étude des aspects médicaux associés à la latence diagnostique permettrait d’établir une sensibilisation et une formation continue pour les médecins et personnels soignants présents en première ligne chez ces patients à risque de négliger des pathologies graves et ainsi d'optimiser le circuit de prise en charge du cancer du sein.
